# Supplementary material for: A Critical View of the Application of the APEX Software (Aqueous Photochemistry of Environmentally-Occurring Xenobiotics) to Predict Photoreaction Kinetics in Surface Freshwaters
Source: Molecules. 2019 Dec 18;25(1):9. doi: 10.3390/molecules25010009 (PMC7017383; doi:10.3390/molecules25010009)
Supplement: Supplementary file 1 [file molecules-25-00009-s001.zip › 3.2.4_gcc-4.4.0.zip!/3.2.4_gcc-4.4.0/3.2.4_gcc-4.4.0/doc/octave/html/interpreter/Matrix-Manipulation.html]

Matrix Manipulation - Untitled


Next: Arithmetic,
Previous: Plotting,
Up: Top


---

## 16 Matrix Manipulation

There are a number of functions available for checking to see if the
elements of a matrix meet some condition, and for rearranging the
elements of a matrix. For example, Octave can easily tell you if all
the elements of a matrix are finite, or are less than some specified
value. Octave can also rotate the elements, extract the upper- or
lower-triangular parts, or sort the columns of a matrix.

- Finding Elements and Checking Conditions- Rearranging Matrices- Applying a Function to an Array- Special Utility Matrices- Famous Matrices
